# Pruebas de vida, pruebas de muerte: Antropología del cáncer entre docentes rurales expuestas a agroquímicos en el sudeste de Córdoba (Argentina)

**DOI:** 10.18294/sc.2023.4442

**Published:** 2023-07-14

**Authors:** Lucía Caisso

**Affiliations:** 1 Doctora en Ciencias de la Educación. Investigadora Asistente, Consejo Nacional de Investigaciones Científicas y Técnicas con sede en Centro de Investigaciones y Transferencia Rafaela, Universidad Nacional de Rafaela, Santa Fe, Argentina. lucia.caisso@gmail.com Consejo Nacional de Investigaciones Científicas y Técnicas Universidad Nacional de Rafaela Centro de Investigaciones y Transferencia Rafaela Consejo Nacional de Investigaciones Científicas y Técnicas Santa Fe Argentina lucia.caisso@gmail.com

**Keywords:** Antropología, Agroquímicos, Cáncer, Docentes, Argentina, Anthropology, Agrochemicals, Cancer, Teachers, Argentina

## Abstract

En este artículo se presentan resultados de una investigación antropológica sobre el cáncer entre docentes rurales expuestas ocupacionalmente a agroquímicos. El estudio se desarrolló en la zona sudeste de la provincia de Córdoba (Argentina), caracterizada por la producción a gran escala de cultivos transgénicos tratados de manera intensiva con plaguicidas agrícolas. A nivel metodológico, el trabajo de campo realizado entre 2019 y 2020 incluyó entrevistas en profundidad a diez docentes y observación de situaciones de la vida cotidiana en los poblados donde vivían y trabajaban estas docentes. Se propone como hallazgo principal que existe una narrativa hegemónica que naturaliza e invisibiliza la existencia del cáncer pero que, a pesar de ella, es posible documentar los padecimientos individuales y sociales que esta enfermedad provoca entre las docentes rurales. Se concluye que es necesario visibilizar esos padecimientos para resguardar la salud y la vida de este sector de la docencia argentina.

## Abstract (en)

This article presents the results of anthropological research on cancer among rural teachers occupationally exposed to agrochemicals. The study was carried out in the southeastern region of the province of Cordoba (Argentina), an area characterized by the large-scale production of transgenic crops intensively treated with agricultural pesticides. Regarding the methodology, fieldwork was conducted between 2019 and 2020 and included in-depth interviews with ten teachers, as well as observations of everyday situations in the towns where they live and work. Among the main findings, it was possible to identify a hegemonic narrative that naturalizes the existence of cancer and renders it invisible; despite this, it was possible to document the social suffering it caused among rural teachers. The article concludes that there is a need to bring visibility to these conditions in order to protect the health and wellbeing of this sector of Argentine teaching professionals.

## INTRODUCCIÓN

“Yo le pregunto, señor gobernador: ¿los docentes y alumnos rurales seguiremos indefensos ante las fumigaciones? […] Es mi deber como maestra tener esperanza de que mis alumnos y los demás niños del país conocerán un mundo mejor” *Estela Lemes. Carta abierta al Sr. Gobernador de Entre Ríos*

Desde hace algunos años me he interesado en la problemática de las fumigaciones con agroquímicos en el entorno de escuelas rurales de la pampa húmeda argentina. Se trata de un interés que ha surgido a propósito del desarrollo de luchas sociales que denuncian los efectos sanitarios y ambientales del uso intensivo de estos químicos -llamados críticamente “agrotóxicos”- y que demandan el cese o al menos una regulación más restrictiva para su uso^1^^,^^2^^,^^3^^,^^4^. Estos procesos de lucha han dado lugar, en determinados puntos de Argentina, al surgimiento de colectivos docentes organizados en torno al problema, tales como la Red Federal de Docentes por la Vida^5^^,^^6^, y al desarrollo de iniciativas sindicales docentes orientadas a su abordaje como, por ejemplo, el protocolo de actuación ante las prácticas de pulverización en entornos escolares, elaborado por la Confederación de Trabajadores de la Educación de la República Argentina (CTERA)^7^. 

El trabajo de campo se llevó a cabo, en un primer momento, en escuelas del centro-norte de la provincia de Córdoba, luego en el sudeste de este territorio provincial y, actualmente, se localiza en escuelas del centro-oeste de la provincia Santa Fe. Si bien la investigación buscó abordar de manera general el problema de las fumigaciones en los entornos escolares, me enfoqué particularmente durante un tiempo específico -y a causa de una beca que me otorgó el Instituto Nacional del Cáncer de Argentina- en el análisis de la relación *plaguicidas-docentes rurales-cáncer*. El artículo que presento en esta ocasión condensa los principales resultados de la investigación desarrollada en el marco de esa beca y basada en el trabajo de campo que desarrollé en la zona sudeste de la provincia de Córdoba (específicamente en los departamentos Unión y Marcos Juárez). 

Dado que la perspectiva disciplinar desde la cual desarrollé la investigación ha sido antropológica/etnográfica las preguntas que orientaron el estudio giraron en torno a qué sentidos construyen las personas docentes expuestas a plaguicidas en torno al cáncer: ¿se sienten especialmente afectadas por esta enfermedad? ¿La relacionan con la exposición a los plaguicidas agrícolas? ¿Experimentan, individual o colectivamente, padecimientos vinculados al cáncer? No obstante, me pregunté también si, más allá de documentar la perspectiva de las personas con relación al tema, era posible reconstruir etnográficamente la existencia/inexistencia de cáncer entre ellas.

En la búsqueda por construir posibles respuestas a estos interrogantes se fue desplegando la investigación que presento en los próximos apartados. En el primero de ellos, recupero los aportes de otras investigaciones que han abordado la problemática de las pulverizaciones con plaguicidas agrícolas en contextos rurales, así como también las contribuciones teóricas con las que dialogué a lo largo del estudio. En el segundo apartado, realizo especificaciones acerca de la metodología empleada para reconstruir informaciones y preciso las características generales que adquieren, a nivel metodológico, las investigaciones antropológicas/etnográficas. En el tercer apartado, analizo la invisibilización social que opera respecto de la posible relación *exposición a plaguicidas* y *cáncer*; invisibilización que registré tanto a nivel de los obstáculos con los que me encontré al inicio de mi investigación como a propósito de algunos sentidos de las docentes rurales entrevistadas. En el cuarto apartado, abordo ciertos padecimientos de las maestras rurales, a propósito del cáncer, que fue posible reconstruir a partir del trabajo de campo y a pesar de los procesos de invisibilización sobre la temática. Por último, presento algunas reflexiones finales sobre el análisis desplegado previamente y propongo la importancia de fortalecer estudios que permitan visibilizar la relación entre agroquímicos, cáncer y docencia rural en Argentina.

### Antecedentes y perspectiva teórica

Entre las investigaciones sociales, que a nivel nacional y regional han indagado sobre el uso de plaguicidas agrícolas, pueden citarse trabajos antropológicos como los de Diez^8^, Lucero^9^ Kunin y Lucero^10^, Caisso^11^^,^^12^, Evia^13^^,^^14^, Kretschmer, Areco y Palau^15^ o González^16^, entre otras. También, desde vertientes más sociológicas o filosóficas se han analizado los procesos de movilización y lucha colectiva a través de los cuales se demandan regulaciones a las pulverizaciones de plaguicidas y se impugnan sus efectos sanitarios y ambientales^1^^,^^3^^,^^17^^,^18^,^^19^^,^^20^^,^^21^. A nivel internacional la problemática ha sido abordada en trabajos como Galt^22^, Kannuri y Jadhav^23^, Bureau-Point^24^, Shattuck^25^ o Grandia^26^. 

Una lectura global de estos estudios evidencia que -más allá de sus particularidades- la indagación sostenida entre sujetos y colectivos, que utilizan cotidianamente plaguicidas agrícolas o que están expuestos de manera sostenida a ellos, permite tomar distancia de la imagen socialmente extendida de campesinos, trabajadores y pobladores rurales como “ignorantes” de los riesgos para la salud que suponen estos químicos. Por el contrario, estas investigaciones reconstruyen experiencias, significaciones y saberes que muestran a las personas expuestas como sujetos activos cuya agencia se manifiesta de diversas maneras que incluyen la producción de saberes legos acerca de la toxicidad de los plaguicidas, el autocuidado o el cuidado colectivo, las críticas y resistencias cotidianas o procesos más excepcionales de movilización colectiva a propósito de este problema. 

Al mismo tiempo, estas investigaciones evidencian que los procesos de invisibilización y/o la naturalización de los padecimientos asociados al uso de plaguicidas deben ser interpretados en términos de las relaciones de dominación que experimentan los sujetos a nivel individual y comunitario. Así, el miedo a perder el empleo, a la estigmatización social en un medio productivo que es plaguicida-dependiente o la falta de acceso a elementos básicos de protección para garantizar el cuidado propio y ajeno no pueden ser soslayados en el marco de los análisis. Del mismo modo, los estudios enfatizan el papel que cumplen las agencias internacionales, las empresas y los Estados (e incluso la relación imperialista de unos Estados sobre otros) en la invisibilización de los riesgos sanitarios del uso de plaguicidas. Esto se produce a partir de la presión comercial, del ocultamiento o no producción de información epidemiológica de importancia o de la presentación engañosa de la información ante los productores, los consumidores o la población en general. 

Otro conjunto de antecedentes de importancia para esta investigación fueron los estudios epidemiológicos críticos que han abordado la relación agroquímicos-cáncer en Argentina. Me refiero a investigaciones como Álvarez^27^, Pujol^28^, Ávila Vázquez y equipo^29^, Delgado^30^ o Verseñazzi *et al*.^31^. Estos trabajos han mostrado -específicamente para la provincia de Córdoba y también para la provincia de Santa Fe, con una misma orientación productiva- que las tasas de muerte por cáncer se encuentran incrementadas en las localidades situadas en contextos productivos caracterizados por el uso intensivo de plaguicidas agrícolas. A estos estudios epidemiológicos se suman las investigaciones de genotoxicidad que evidencian que la exposición ocupacional a plaguicidas agrícolas incrementa las aberraciones cromosómicas que pueden, en el futuro, favorecer el desarrollo de tumores^32^^,^^33^.

Un tercer grupo de antecedentes relevantes para esta investigación son los estudios sociológicos y antropológicos sobre *cáncer* que abordan las distintas dimensiones -políticas, afectivas, emocionales, familiares- implicadas en la experimentación subjetiva y cotidiana del padecimiento asociado a esta enfermedad. Me refiero a investigaciones como las de Garassini^34^, Suárez Rienda y López Sánchez^35^, Porroche Escudero^36^, y Passerino^37^^,^^38^. Estos trabajos me permitieron abordar de manera más compleja, profunda y situada el modo en que los sujetos y sus afectos transitan el cáncer (o la muerte asociada a él). Me sirvieron, además, para repensar mi historia con relación al cáncer de mama y ejercer la necesaria reflexividad antropológica sobre ese aspecto de mi propia vida. 

Por último, con relación a las contribuciones teóricas con las cuales dialogué a lo largo del estudio, me interesa recuperar los aportes de Antonio Gramsci que fueron sintetizadas para el campo antropológico por Crehan^39^^,^^40^ y Roseberry^41^. Desde este marco referencial, las significaciones culturales no son entendidas *como un horizonte estático y esencial de referencia sino como un conjunto de prácticas y sentidos que se encuentran permeados por las disputas sociales en desarrollo.* Interpretar las significaciones y prácticas culturales en torno a los *fenómenos ambientales que afectan la salud* supone una reconstrucción de la relación dialéctica entre *relaciones de dominación y agencia/resistencia*. Entiendo que es en este mismo sentido que se señala, desde el campo de la antropología de la salud y de la epidemiología sociocultural, la importancia de atender a las relaciones sociales más generales que configuran los procesos de salud/enfermedad/atención^42^^,^^43^ y se enfatiza la necesidad de atender a la ética productivista del capitalismo como elemento de importancia capital en la génesis de los problemas sanitarios^44^. De esta manera, se toma distancia de las concepciones individualizadas, biologicistas y ahistóricas que sobre esos procesos han construido los enfoques biomédicos y positivistas y el modelo médico hegemónico^45^^,^^46^. 

## METODOLOGÍA DE LA INVESTIGACIÓN

En términos metodológicos esta investigación se desarrolló a partir de un enfoque etnográfico. Rockwell^40^ propone que la *etnografía* no es un conjunto de técnicas, sino una perspectiva particular de abordaje, la cual reúne como principales características: 1) la etnógrafa y el etnógrafo se constituyen como *cronistas de lo no documentado* de la realidad social (lo familiar, lo cotidiano, lo invisibilizado) para dejar testimonio escrito y público de ello; 2) el producto del trabajo es ante todo *una descripción* que busca reflejar la complejidad de la realidad observada y que se sustenta *en categorías teóricas*; 3) quien investiga y su *experiencia directa* tienen un papel central en la investigación, generando vínculos con los sujetos de estudio y realizando, al mismo tiempo, tanto las tareas de reconstrucción de información como el análisis; 4) se da relevancia a los significados, saberes y explicaciones sobre los acontecimientos sociales que poseen los sujetos estudiados; 5) a partir de la descripción y análisis de realidades particulares, se construye un conocimiento significativo para las inquietudes teóricas y prácticas más generales^47^. El valor de desarrollar estudios etnográficos de esta clase, sobre problemas de salud colectiva, reside en que pueden aportar informaciones ricas, complejas y contextualizadas que suelen no ser tenidas en cuenta en el marco de las reconstrucciones epidemiológicas estadísticas^48^.

El trabajo de campo de esta investigación se realizó entre los años 2019 y 2020 en distintas ciudades pequeñas de los departamentos Unión y Marcos Juárez del sudeste de la provincia de Córdoba, en el centro geográfico de la Argentina. Se entrevistaron a diez personas, todas docentes, de las que se obtuvo el consentimiento informado y a las que se otorgó la hoja de información aprobada por el Comité Institucional de Ética en Investigación en Salud del Hospital Nacional de Clínicas de Córdoba (Libro de Actas II, nº194, 23/04/2020). No obstante, en algunas ocasiones las entrevistas contaron también con la presencia de otras personas (docentes, vecinas y vecinos o familiares) que fueron consultadas por la persona que estaba siendo entrevistada y que contribuyeron con informaciones relevantes para la investigación. Casi todas las personas entrevistadas resultaron ser mujeres en virtud de la histórica feminización del sector docente en la República Argentina^49^^,^^50^. Por este motivo, utilizaré en este texto, y de aquí en más, artículos y desinencias femeninas para referirme a las personas que entrevisté. 

La selección de las docentes entrevistadas siguió dos criterios: que fueran maestras de escuelas rurales de nivel primario y que sus escuelas se encontraran en los departamentos de Unión y Marcos Juárez. La elección de docentes de nivel primario respondió al hecho de que las instituciones de este nivel educativo se encuentran ubicadas en ámbitos rurales más dispersos (es decir, más alejados de ciudades o pueblos) que, por ejemplo, las escuelas de nivel secundario. Por este motivo las considero como instituciones más paradigmáticamente expuestas a las pulverizaciones con plaguicidas, lo que no significa que los centros educativos de otros niveles no estén también expuestos a estos químicos. La selección de la zona de trabajo de las docentes entrevistadas se fundamentó en el hecho de que los departamentos de Unión y Marcos Juárez integran una zona mundialmente reconocida por la producción de cultivos transgénicos^51^, los cuales son pulverizados de manera sistemática con plaguicidas agrícolas. 

Algunas de las docentes entrevistadas tenían entre 40 y 55 años de edad y entre 15 y 25 años de antigüedad en la docencia rural. Otras, más jóvenes, habían iniciado hacía pocos años atrás su trayectoria laboral. Los encuentros se realizaron en sus casas particulares, no duraron más de una hora y media cada uno. Las conversaciones fueron audiograbadas y posteriormente transcriptas. 

Las primeras preguntas de las entrevistas giraron en torno a los interrogantes iniciales de la investigación, vinculados a: las condiciones de exposición ocupacional de las docentes a las “fumigaciones” con agroquímicos; la existencia/no existencia de cáncer entre ellas; su percepción sobre los efectos de los plaguicidas sobre su salud. No obstante, y dado el carácter no directivo que poseen las entrevistas etnográficas^52^, los encuentros con las entrevistadas se abrieron a la indagación de otros elementos o dimensiones que no habían sido contemplados previamente y que constituyeron inesperadas claves de acceso a sus propios modos de entendimiento acerca del problema de estudio. Además, como veremos en el cuarto apartado, respecto de una pregunta que era respondida en primera instancia de una manera, podían restituirse luego -a propósito de la conversación sobre otras cuestiones- otras informaciones diferenciadas o incluso contradictorias.

En el marco de la investigación, la centralidad que adquirió la estrategia metodológica de la entrevista se debió, en gran medida, a la imposibilidad de realizar observaciones participantes dentro de las escuelas en las que trabajan las docentes, dado que las autoridades del Ministerio de Educación de la provincia de Córdoba que debían evaluar mi pedido de autorización para realizar trabajo de campo me negaron dicho acceso, cuestión sobre la que volveré en el próximo apartado. Para solventar el obstáculo metodológico que supuso no poder realizar observaciones en las escuelas, solicité a las docentes que confeccionaran mapas artesanales de sus escuelas y sus contextos productivos y ambientales. Algunas de ellas aportaron posteriormente fotografías y videos con los que ilustraron aquello que me habían relatado durante las entrevistas o lo que habían dibujado en sus mapas. También confeccioné -como en el marco de cualquier otro estudio etnográfico- registros de campo de mis propias vivencias en los poblados y ciudades de la zona de estudio, en tanto estos son también los sitios en los que viven las docentes que trabajan en las escuelas rurales de la región. 

Con relación al análisis del material producido en el marco del trabajo de campo, cabe señalar la naturaleza dialéctica de la relación entre este y el trabajo conceptual en el marco de las investigaciones antropológicas. Tal como señala Achilli^53^ el trabajo de campo y su análisis conforman un proceso simultáneo a través del cual se van generando distintas producciones escritas, tendientes a relacionar fragmentos de información empírica y ponerlas en diálogo con los antecedentes de investigación y con los aportes teóricos retomados. Es en ese diálogo continuo -y siempre provisional e inconcluso- que construimos ejes analíticos que permitan aprehender algunos de los procesos propios de la realidad estudiada. En el marco de esta investigación, esos ejes analíticos se vincularon, por un lado, a la *invisibilización de la relación plaguicidas-cáncer* (como expresión de las relaciones de dominación) y, por el otro, a la *existencia de padecimientos asociados al cáncer entre las maestras rurales* (y a la posibilidad de su reconstrucción como expresión de la agencia individual y colectiva de los sujetos). Los apartados de este artículo que corresponden a la presentación de resultados se han estructurado, por tanto, a partir de dichos ejes de análisis. 

Por último, cabe aclarar que todos los nombres propios que aparecen en este artículo son seudónimos. Por esta vía se busca proteger la identidad real de las mujeres que han participado de esta investigación a fin de no exponerlas públicamente. En el caso de las docentes fallecidas o enfermas por cáncer se ha preservado el nombre real de pila, aunque se omiten tanto sus apellidos como las instituciones educativas en las que trabajan o trabajaron.

## RESULTADOS

### *“Yo no soy médico*”: La invisibilización de la asociación plaguicidas-cáncer entre las docentes rurales

Al igual que en el resto de la pampa húmeda argentina, se han introducido en el sudeste de Córdoba, desde la década de 1990, cultivos de soja y maíz modificados genéticamente. Estos cultivos son pulverizados al menos tres veces por año, tanto de manera aérea con avionetas fumigadoras, como de manera terrestre con máquinas fumigadoras autopropulsadas conocidas como “mosquitos”. Los plaguicidas utilizados son diversos, aunque aquellos cuyo principio activo es el glifosato lideran las ventas. Además, estos plaguicidas se mezclan entre sí de diversas formas y son aplicados en cantidades crecientes año a año en búsqueda de una mayor eficiencia en la batalla con las plagas, que en cada campaña agrícola se vuelven más resistentes, en virtud de la selección natural^54^.

Algunos pocos años antes de iniciar esta investigación, se había producido en Córdoba un evento de importancia, vinculado a la problemática que me disponía a investigar: la publicación, en 2014, de los datos del Registro de Tumores a nivel departamental, que actualmente solo se encuentran disponibles a nivel provincial^55^. Según los datos desagregados, los departamentos Unión y Marcos Juárez, así como también otros departamentos provinciales dedicados a la agricultura intensiva, poseían las tasas de mayor existencia de cáncer a nivel provincial. Esta información se dio a conocer de manera masiva el día 29 de mayo de 2014 a través de una noticia publicada en *La Voz* (el diario cordobés de mayor tirada), bajo el título “El mapa del cáncer en Córdoba”^56^. 

El impacto social que provocó esta publicación fue considerable, en tanto presentó evidencias epidemiológicas de los argumentos sostenidos desde hacía casi dos décadas por los colectivos vecinales y socioambientales que venían denunciando los efectos sanitarios de las fumigaciones: denuncias que se apoyaban en los resultados de algunas de las investigaciones críticas que se reseñaron en el primer apartado y que ya se habían realizado por aquel entonces, como las de los equipos de Ávila Vázquez^29^, Álvarez^27^, Pujol^28^, y Peralta *et al*.^32^. Los comunicados, nuevas notas periodísticas y hasta movilizaciones en la ciudad de Córdoba capital no se hicieron esperar en los días posteriores a la publicación del “mapa”^56^. Finalmente, y presumiblemente bajo presión de las corporaciones públicas y privadas del agro, el periódico cordobés terminó por eliminar la nota de su sitio web durante un lapso prolongado -hoy se encuentra nuevamente disponible^56^- y el Registro Provincial de Tumores dejó de desagregar la información epidemiológica por departamentos. No obstante, todavía puede encontrarse una reproducción de este mapa en Barri^57^ y la reconstrucción de los hechos en torno a la publicación del “mapa del cáncer” en Sández^58^.

La polémica desatada en torno al “mapa del cáncer” delineaba un escenario controversial en torno a la problemática que me disponía a estudiar, y se aunaba a la carencia de información epidemiológica oficial que me permitiera correlacionar la exposición ocupacional a plaguicidas de la docencia rural con la existencia de cáncer. Este vacío se originaba, en primer lugar, en la inexistencia de datos sobre cáncer que desagregaran la variable ocupacional de las personas encuestadas. En segundo lugar, se trataba de un vacío provocado por el subregistro de enfermedades profesionales que presenta el sector docente, tanto en ámbitos rurales como urbanos^59^. Esto se sumaba, además, al subregistro de enfermedades profesionales y accidentes de trabajo vinculados a la intoxicación aguda o a la exposición crónica a plaguicidas, problemática reconocida como grave, incluso en informes emitidos desde organismos estatales de nuestro país^54^^,^^60^. Las investigaciones independientes, por su parte, si bien habían analizado el efecto carcinogénico y/o mutagénico de la exposición ocupacional a los plaguicidas o el incremento de las muertes por cáncer en las zonas rurales caracterizadas por su uso, no habían indagado específicamente en la salud de docentes rurales expuestos a estos químicos. 

Un tercer obstáculo lo constituía, desde el punto de vista de la salud ocupacional, la falta de jurisprudencia que permitiera reconocer y encuadrar los problemas sanitarios derivados de la exposición ocupacional docente a plaguicidas como enfermedades profesionales, cuyo caso más emblemático es el de la docente rural Estela Lemes^61^. Al entrevistarme con la secretaria de Salud, Medioambiente y Trabajo del gremio que nuclea a los docentes de Córdoba me relató que al menos dos docentes rurales se habían acercado a la entidad buscando asesoramiento jurídico a causa de enfermedades que padecían y que vinculaban a su exposición a los plaguicidas. Sin embargo, la entidad gremial no había podido “dar curso al reclamo” porque, dado que las docentes no manipulan ellas mismas los plaguicidas a propósito de su actividad laboral, consideraban que resultaría muy difícil que la aseguradora de riesgos del trabajo la reconociera como enfermedad ocupacional. *“Además viste cómo es… es un tema muy vidrioso… difícil de tratar”* agregaría la mujer para concluir nuestra charla. 

La respuesta del Ministerio de Educación de la provincia de Córdoba a mi requerimiento de autorización para llevar adelante mi estudio ingresando a las escuelas resultó ser la confirmación de que aquello que decía la secretaria de Salud del gremio docente -y más allá de mi valoración sobre el tratamiento del tema por parte de la entidad gremial- era cierto: se trataba de un tema “*difícil de tratar*” y, por lo tanto, de estudiar. Los funcionarios ministeriales con los que busqué obtener la autorización no se negaron explícitamente a otorgarla, sino que me pidieron que trasladara mi pedido “*al Ministerio de Agroindustria*”. Consideré este mensaje como una declaración estatal acerca del poder que detenta el agronegocio en la provincia de Córdoba. 

Pero la ocultación de datos que previamente habían estado disponibles (el mapa del cáncer o los datos de muertes por tumores desagregados por departamentos), el subregistro de enfermedades profesionales, las limitaciones jurídicas para reconocer estas enfermedades como profesionales e, incluso, las dificultades para comenzar a realizar la investigación constituían, antes que obstáculos en su desarrollo, parte de la problemática investigada. Como también señalan respecto de sus propias investigaciones Lucero^62^ y Delgado^30^ se trata de elementos contextuales que constituyen evidencias de la invisibilización social que se opera en torno a la relación agroquímicos-salud y su potencial vinculación con la existencia de cáncer. 

Estos elementos, sin embargo, no conforman un escenario externo de grandes relatos disociados de la vida cotidiana de los sujetos de estudio. A partir del trabajo de campo, pude reconocer la presencia de estas invisibilizaciones en la mayoría de los sentidos de las docentes acerca del tema de las pulverizaciones y pese al reconocimiento que ellas realizan del cáncer como una realidad sanitaria extendida en la zona:

“*Bueno cáncer hay, sí… Mi papá ha fallecido de cáncer en el cerebro ¿no?... sí, se escucha mucho, mucho… de niños también. Pero bueno… no que puedas decir ‘es por la fumigación’* [...] *Yo viajo dos veces a la semana con una médica de Corral de Bustos… le conté que vos me ibas a entrevistar por este tema y le pregunté si ella notaba que las fumigaciones afectaran* [a la salud] *y me dijo que no… no… Sí ha tenido, dice, algún caso aislado, pero porque… por descuido de la persona digamos… como, por ejemplo, que se ha metido adentro de un silo sin protección, o por haber estado fumigando sin algún tipo de protección. Pero que ella no nota que se hayan dado casos de…bueno* [cáncer]… *por este motivo*”(Entrevista a Inés, docente rural en la zona de influencia de la ciudad de Corral de Bustos, noviembre de 2019)

“*Hubo varios casos de gente con cáncer… ahora falleció hace poquito un chico creo que de leucemia. Después hubo un muchacho que tuvo leucemia pero que se curó, era jovencito. Bueno, cáncer de mama… por ejemplo, mi vecina, cuarenta y pico de años, se curó. Después conozco un caso de una chica de treinta y pico de años, que hace muchos* a*ños… debe hacer ya como veinte años que falleció de cáncer de mama. Bueno, también mi suegro, de próstata. Murió hace seis años, a los sesenta y nueve. Pero siempre así algo puntual, digamos… y no de gente que trabaje fumigando*”(Entrevista a Virginia, exdocente rural en la zona de influencia de la ciudad de Guatimozín, diciembre de 2019)

“*Hay muchos, hay muchos* [casos de cáncer]… *Justamente el domingo estaba en el partido, fui a ver el club de acá y había fallecido de cáncer el suegro de una chica que estaba al lado mío. Y hablando con ella me dice… ‘sí, la suegra de mi hermana también* [falleció por cáncer]’… *‘sí, y el marido de la chica que es bombera también’… O sea, se da el tema del cáncer en… ¡en jóvenes! ...Y leucemia en niños… Yo no sé con qué tiene o no que ver, yo no soy médico… Hay gente que te dice que son las fumigaciones, pero yo tengo mi yerno que es ingeniero agrónomo… le conté a él que me ibas a entrevistar y me dice: ‘la gente está en contra del glifosato, pero el glifosato es etiqueta verde que es la permitida. Hay otras etiquetas peores como el Off y la gente se lo pone* [igual]*… o etiqueta azul el Raid’. Me dice ‘te lo voy a anotar’… y justamente le digo: ‘sí… anotame porque esta chica me va a preguntar’…”*(Entrevista a Esther, docente rural en la zona de influencia de la ciudad de Corral de Bustos, diciembre de 2019)

En estos relatos, que ilustran también otros testimonios reconstruidos a partir de las entrevistas a las docentes, se torna evidente, por un lado, que el cáncer forma parte de la vida cotidiana de los poblados rurales en los que viven estas mujeres y que están próximos a las escuelas en las que trabajan. Sin embargo, la pregunta por la posible asociación entre esta enfermedad y las pulverizaciones con plaguicidas aparece, de distintos modos, cancelada u obturada: Esther no la puede contestar porque *no es médico* y porque su yerno, que es ingeniero agrónomo, desestima dicha asociación; Virginia la desestima porque el cáncer -aunque en su relato contabiliza varias víctimas- constituye casos “puntuales” que no están vinculados a la manipulación directa de plaguicidas; Inés busca la opinión de una profesional -una médica amiga- que descarta la asociación y vincula los efectos sanitarios de los plaguicidas con las intoxicaciones agudas ocasionadas por conductas laborales inapropiadas (tales como no utilizar protección al ingresar un silo o realizar una práctica de pulverización). 

En estos sentidos de las docentes es posible reconocer, entramados, los argumentos centrales de la narrativa hegemónica sobre la cuestión agroquímica. Así, por ejemplo, la centralidad que adquieren las opiniones de la médica y del ingeniero agrónomo en el relato de Esther y de Inés puede vincularse con el argumento hegemónico relevado por Folguera^20^, Gárgano^21^ y González^16^, que erige como los únicos saberes legítimos para participar de la controversia sobre los efectos de los plaguicidas a las opiniones de técnicos y profesionales. Se desconocen por esta vía tanto los saberes que producen los sujetos afectados en el marco de procesos de movilización social, como también los estudios científicos críticos que los avalan, tal como los ya referidos de Peralta *et al*.^32^, Butinof *et al*.^33^, Álvarez^27^, Pujol^28^, Delgado^30^, Verseñazzi *et al*.^31^, entre otros. 

Por otro lado, con relación a la asociación exclusiva entre enfermedad y manipulación directa de agroquímicos (y, por lo tanto, trabajo rural “mal practicado”) Löwy^19^, Shattuck^25^ y Caisso y Carreño^63^ señalan cómo los intereses empresariales, articulados especialmente en torno al discurso de “buenas prácticas agrícolas”, proponen que son los sujetos trabajadores rurales -y no la matriz productiva dependiente de estos químicos- los agentes responsables de los efectos sanitarios adversos que una “mala práctica agrícola” pueda provocar. De esta manera, al centrarse el análisis en la responsabilidad individual de la persona usuaria de plaguicidas, se oscurecen las causas estructurales que subyacen a su utilización y, por lo tanto, sus efectos. Además, en los relatos citados de las docentes, se asocian los efectos sanitarios de los plaguicidas solo a su exposición aguda (es decir, a los efectos del contacto directo con el plaguicida) sin considerar la “lenta violencia” de impactos sobre la salud que pueden provocar estos químicos^26^.

**Figura 1:**
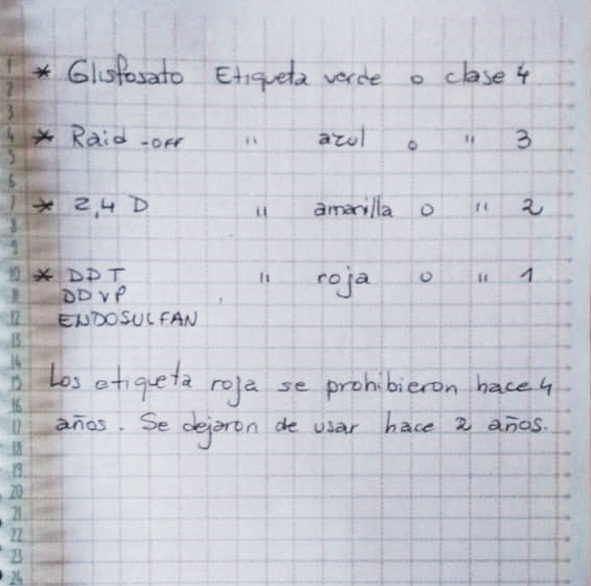
Listado de plaguicidas e insecticidas agrícolas y hogareños elaborado por un ingeniero agrónomo y yerno de una de las docentes entrevistadas. En él se indica la “clase” de peligrosidad de cada uno de estos productos. Santa Fe, Argentina, 2020

Por último, la tabla que ha escrito el yerno de Esther a propósito del encuentro de la mujer conmigo (Figura 1) puede ser interpretada como parte de las acciones pedagógicas o formativas de los sectores dominantes del agro que releva González^16^. Se trata de una serie de acciones que evidencian un cambio de modalidad en la comunicación empresarial: si antes, frente a los cuestionamientos sociales a los plaguicidas, la narrativa hegemónica se articulaba fundamentalmente en torno a operaciones de silencio social -tal como relevamos en Caisso^11^- en la actualidad, se despliega más bien en torno a lenguajes activos que buscan “comunicar” la seguridad de los plaguicidas cuando son utilizados en el marco de “buenas prácticas agrícolas”. 

En este sentido, persiguiendo instruirnos acerca de la no peligrosidad de los plaguicidas agrícolas, el yerno de Esther nos muestra, a través de la tabla que ha confeccionado, que los plaguicidas a base de glifosato serían incluso menos peligrosos que los productos utilizados para combatir moscas y mosquitos: mientras que los primeros son clase IV (o “etiqueta verde”) los segundos son clase III (o “etiqueta azul”). Respecto de esta tabla, hay que decir, en primer lugar, que se trata de una homologación totalmente engañosa, en tanto la exposición a plaguicidas se produce a una escala mucho mayor y mucho más sistemática que la exposición a plaguicidas hogareños (la cual, en todo caso, también podría debatirse socialmente para que se regule de manera más segura). Pero, además, Galt^22^ nos recuerda la trampa de marketing subyacente a la presentación de un plaguicida como “verde”, término asociado a la naturaleza y al movimiento ecologista. Y, puntualmente, respecto de la información brindada en la tabla con relación a la clasificación del glifosato, Löwy^19^ nos alerta acerca del “error” en el que sistemáticamente ha caído el agronegocio, al desconocer en el etiquetado de los plaguicidas la recategorización del glifosato como Clase III, en el año 2012. 

Cuando avanzo a lo largo de las entrevistas sobre la pregunta relacionada con la existencia de cáncer entre las docentes, éstas me dicen que si bien se consideran ocupacionalmente expuestas a los plaguicidas y saben que estos pueden ocasionar efectos adversos sobre la salud (tales como alergias cutáneas, irritación en los ojos y boca o náuseas) no consideran que el cáncer las afecte particularmente a ellas. Y, al menos en una primera instancia -volveremos a este tema en el próximo apartado- dicen no recordar docentes rurales enfermas o fallecidas por cáncer. 

Al obtener esta respuesta me pregunto en qué medida las invisibilizaciones que he reconstruido hasta aquí (incluidos los argumentos de la narrativa hegemónica que aparecen entramados en los sentidos de las docentes) no operan cancelando la posibilidad de reconstruir evidencias de la existencia de cáncer entre las entrevistadas. Reflexiono particularmente sobre esta cuestión a causa de un pequeño diálogo que registro en un consultorio médico en la ciudad de Corral de Bustos, al que asisto a causa de un problema físico que me aqueja. Mientras espero mi turno en la sala de espera, una enfermera conversa con otro paciente: un hombre de mediana edad, que acaba de salir de una sesión de radioterapia con la que trata un cáncer de garganta que lo afecta. La mujer le comenta que su tía también está bajo tratamiento “de rayos” porque tiene cáncer de mama. Como buscando darle ánimos al hombre la enfermera le dice:

“*sé que no es fácil* [atravesar un cáncer]… *pero como le digo yo a mi tía… hacer los tratamientos es una prueba de vida… una prueba que hay que pasar para mostrarle a Dios que uno quiere seguir viviendo*”. (Registro de campo, Corral de Bustos, noviembre de 2019)

Al escuchar esta conversación comprendo mejor las dificultades que supone el estudio de esta problemática. En esta zona, pero posiblemente también en muchos otros contextos sociales -y en virtud de narrativas hegemónicas que invisibilizan los padecimientos sociales- la existencia del cáncer pareciera no ser prueba de enfermedad o muerte sino, más bien, de todo lo contrario: es una prueba de vida. Una prueba de redención en virtud del sacrificio y del sufrimiento que provoca la enfermedad^35^. Las personas que padecen cáncer, antes que indagar en las responsabilidades sociales que han provocado su enfermedad, son responsables de dar a través de su padecimiento pruebas de querer seguir viviendo. Se produce de esta manera una inversión perfecta del interrogante: no me pregunto (no me preguntan) por qué enfermo y muero, sino que me pregunto (y me preguntan) si quiero seguir viviendo. 

Sin embargo, esas narrativas que impregnan los sentidos cotidianos desde los cuales los sujetos significan su experiencia con los plaguicidas y su relación con el cáncer no constituyen un paisaje inamovible de dominación. Como nos recuerda Roseberry^41^, la hegemonía es un proceso problemático y disputado antes que una formación ideológica acabada y monolítica. Por este motivo, siempre pueden reconstruirse informaciones que nos hablan de eventos que ocurren a pesar de los esfuerzos de ocultamiento desplegados desde los discursos y las prácticas hegemónicas. Me detendré en estos eventos en el próximo apartado. 

### “Empezar a hacer cadenas”: Padecimientos por cáncer entre las docentes rurales expuestas a los agroquímicos

Según acabamos de ver, la primera respuesta que las docentes rurales me brindan cuando les pregunto si el cáncer las afecta particularmente a ellas es negativa: no recuerdan compañeras enfermas o fallecidas por cáncer. Sin embargo, a partir del desarrollo de las entrevistas, comienzo a reconstruir que si bien ninguna de mis entrevistadas tiene o ha tenido cáncer, sus vidas se encuentran atravesadas por padecimientos asociados a esta enfermedad: muchas de ellas son maestras que han enviudado prematuramente por cáncer, son huérfanas por cáncer, han perdido personas del vecindario, amistades y familiares por cáncer. Verónica, por ejemplo, quien es docente rural en una escuela próxima a la ciudad de Corral de Bustos (en el departamento de Marcos Juárez), al momento de la entrevista, se encuentra de licencia laboral por la muerte de su marido:

“*Yo tenía el cáncer como muy alejado… es cierto que mi mamá y algunas amigas habían tenido, no es que me pegaba de costado el tema. Pero cuando mi marido se enfermó fue distinto… no había forma de remisión… se fue en pocos meses y ahí me fui enterando que en Corral de Bustos hay un montón de gente que sufre de melanoma… no todos de ese tan agresivo pero… pareciera que es toda la zona* […] *Mi marido trabajaba colocando transformadores de EPEC* [Empresa Provincial de la Energía de Córdoba] *en el medio del campo… tenés lo que tienen los transformadores adentro, tenés las fumigaciones… por eso yo no quiero empezar a hacer cadenas* [hacer asociaciones] *porque si empezás* [a hacer asociaciones] *todo te lleva a un mismo lugar*…” (Entrevista a Verónica, maestra rural de la zona de influencia de la ciudad de Corral de Bustos, diciembre de 2019)

Como vemos, además del padecimiento de Verónica con relación a la muerte fulminante de su marido a causa de un melanoma, su relato señala que la experimentación de ese padecimiento la puso en alerta con relación a una situación sanitaria que había pasado, hasta entonces, inadvertida para ella (la existencia de otras personas enfermas o fallecidas por ese tipo de cáncer en la zona). Al mismo tiempo, Verónica vincula los posibles desencadenantes de esas enfermedades y muertes a la exposición a ciertas sustancias tóxicas, tales como los bifenilos policlorados que se utilizan para la refrigeración de los transformadores eléctricos o los plaguicidas agrícolas que son pulverizados en los campos. Sin embargo, prefiere no “*empezar a hacer cadenas*”, es decir, no hacer asociaciones que, según estima, la conducirían a “*un mismo lugar*”. Tal vez a señalar causas cuyo cuestionamiento se representa, al menos por ahora, como una tarea difícil de efectuar. 

La historia de Verónica presenta puntos de contacto y puntos de distancia con la historia de Lidia, quien es docente en la zona de influencia de Laborde y quien también ha enviudado prematuramente. El marido de Lidia, que había trabajado varios años en un negocio de venta de maquinaria agrícola rodeado de campos de cultivo, falleció a los 39 años a causa de un tumor cerebral. *“Es como que la vida se te parte en dos”,* me dice la docente al mismo tiempo que hace un ademán como cortando el aire con una mano, ilustrando que la intrusión del cáncer es vivida por las personas enfermas y quienes las cuidan como un hito o quiebre biográfico^34^. Al igual que Verónica, Lidia también rememora que fue a raíz de la enfermedad y muerte de su marido que comenzó a advertir que muchos otros vecinos y vecinas de su localidad -situada en el departamento Unión- enfermaron y/o murieron por cáncer: gente extremadamente joven, niños y niñas, dos o tres integrantes seguidos de una misma familia. De hecho, dos de los compañeros de trabajo del marido de Lidia murieron el mismo año que él, siendo también muy jóvenes y, ambos, a causa de un cáncer de páncreas. 

Sin embargo, a diferencia de Verónica, Lidia pudo inscribir ese padecimiento individual por la enfermedad y muerte de su marido en un espacio colectivo: una asamblea de vecinos y vecinas que comenzaron a formular preguntas -que empezaron *“a hacer cadenas*”, retomando los términos de Verónica- acerca de las causas por las que la enfermedad y la muerte se expandían de manera tan notable en la localidad. Apoyadas por la municipalidad, las personas llevaron adelante, en articulación con un equipo de investigación de Córdoba capital, un estudio epidemiológico que evidenció la inusual situación sanitaria que se estaba viviendo. Pudo logarse así -aunque no sin sortear numerosas dificultades- una de las regulaciones más restrictivas de “las fumigaciones” en todo el departamento Unión. 

Pero en el devenir de estos relatos de vida que se van desplegando a partir de las entrevistas, van comenzando a surgir -aludiendo a ellas también como pérdidas personales sufridas por las docentes- los nombres de algunas maestras rurales enfermas o fallecidas por cáncer. Porque, a medida que se van hilando recuerdos personales y se va profundizando la conversación, comienzan a recordar algunos casos que aparecen como piezas sueltas y casi al borde del olvido y, por lo general, cuando las entrevistas están por finalizar: *“…me quedé pensando en eso que me preguntabas… y ahora me acuerdo de esta chica que tomó licencia por cáncer…”*, me decía Alejandra, de un modo similar a aquel en el que otras docentes también recordaron al menos uno o dos nombres de colegas enfermas o fallecidas por cáncer antes de que finalicen nuestros encuentros. 

**Figura 2:**
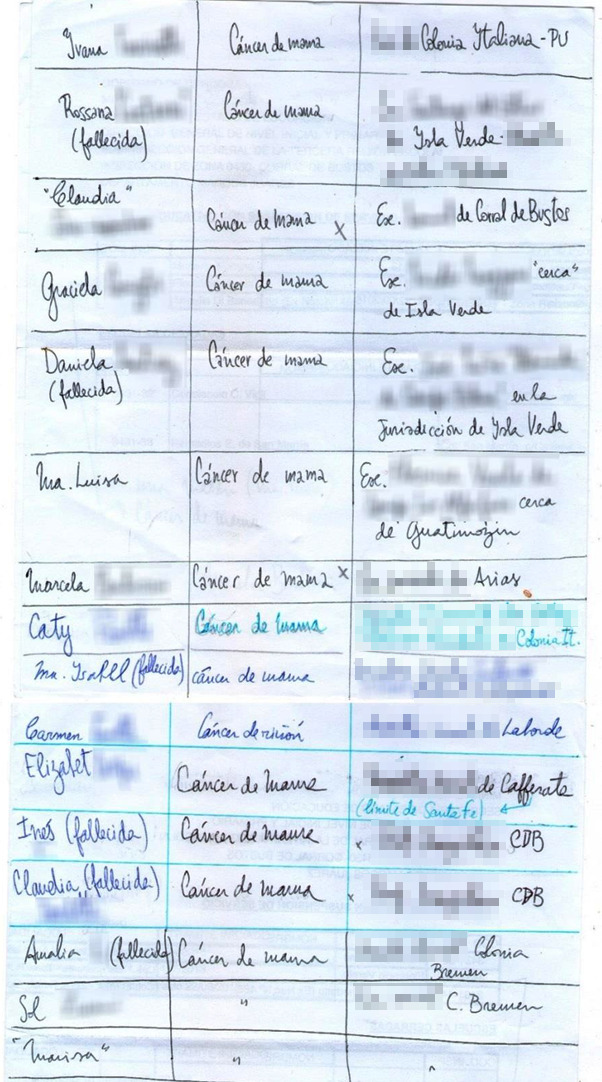
Listado con nombres propios de maestras rurales enfermas o fallecidas por cáncer elaborado a lo largo del trabajo de campo. Se encuentran pixelados los apellidos y nombres de las instituciones educativas donde trabajaban las docentes. Santa Fe, Argentina, 2020.

A lo largo del trabajo de campo voy anotando -aunque sin saber muy bien para qué- estos nombres sueltos en una hoja en blanco. Además, en esa “tabla artesanal”, asocio esos nombres con las escuelas en las que las maestras me dicen que sus colegas enfermas o fallecidas por cáncer trabajan o han trabajado y con el tipo de cáncer que padecen o han padecido. Al finalizar el trabajo de campo mi listado tiene, en total, dieciséis nombres. Dieciséis mujeres de catorce escuelas rurales diferentes. Junto al nombre de quince de ellas puede leerse *“cáncer de mama”* (Figura 2), un tipo de cáncer que ha sido asociado por diversos estudios con la exposición a plaguicidas agrícolas de uso frecuente en Argentina^33^^,^^64^^,^^65^^,^^66^^,^^67^. 

Me pregunto, sin embargo, qué valor puede tener este papel manuscrito y ajado por los vaivenes del trabajo de campo. ¿Constituye realmente una “prueba de muerte” o de enfermedad por cáncer de las maestras rurales expuestas a plaguicidas? Contesto afirmativamente porque, ante la ausencia de estadísticas epidemiológicas oficiales, el lenguaje de los censos y listados artesanales, realizado por personas comunes y solo en segunda instancia refrendado con trabajos epidemiológicos académicos, ha sido el lenguaje común con el que se iniciaron numerosos procesos de lucha por la regulación de las fumigaciones. Así se originaron procesos paradigmáticos para la región de la pampa húmeda, tales como el caso de la localidad de Monte Maíz o del barrio Ituzaingó Anexo en la ciudad de Córdoba. 

Pero, además, al releer cuál es el tipo de cáncer mayoritario que han padecido o por el que han fallecido estas maestras -cáncer de mama- me retrotraigo a mi propia historia de vida: he perdido por un cáncer de mama a mi madre, Analía, cuando ella tenía 43 años y yo solo 15. Al igual que Verónica y Lidia, sé muy bien cómo “*la vida se te parte en dos*” y conozco de cerca la historia de los cuidados, las esperas y las angustias tramadas a nivel familiar. No necesito que me describan cómo es la opresiva sensación en el pecho cuando se acercan las fechas de nuevos estudios y controles, ni el desgaste que supone la búsqueda de todo tipo de terapias que garanticen la anhelada curación. He experimentado incluso, y a temprana edad, la confrontación con la idea de la muerte que se aproxima implacable sobre el ser querido. 

Entiendo entonces, y a partir de la vinculación de mi propia historia personal con este rosario de nombres de las maestras enfermas o fallecidas por cáncer de mama, que el valor del listado no es tanto una prueba cuantitativa de “muerte” o “enfermedad” sino, más bien, un testimonio del padecimiento físico y subjetivo que se teje en torno al cáncer: porque las historias de *Ivana*, de *Rossana*, de ambas *Claudias*, de *Graciela*, de *Daniela*, de *María Luisa*, de *Marcela*, de *Cathy*, de *María Isabel*, de *Elizabeth*, de *Inés,* de *Amalia,* de *Sol* y de *Marisa* seguramente se parecen a la historia de *Analía*, mi madre. Detrás de sus nombres hay una familia, amigas, parejas, colegas y posiblemente también una hija que, como yo, ha sufrido o sufre ante la enfermedad y la pérdida potencial o real de su madre. Detrás de estos nombres se condensa, además, un doble padecimiento: porque sobre los cuerpos que experimentan la enfermedad (e incluso la muerte) recaen también los mandatos-violencias que las relaciones de género imponen a los cuerpos y las vidas de las mujeres^37^^,^^38^. 

Sin embargo, la identificación entre mi propia historia y la de los nombres que componen el listado, no solo me sirve para reflexionar en torno al padecimiento que subyace al cáncer, en general, y al cáncer de mama, en particular. Esa identificación me permite también vislumbrar los mecanismos de aquello que Porroche-Escudero^36^ conceptualiza como la *despolitización del cáncer*. Es decir, su experimentación como evento individual -como una “maldición” personal- desconectado de causas, fenómenos y procesos sociales y ambientales más generales. Sostengo esto en tanto a lo largo de mi historia de vida nunca me había preguntado qué fenómenos ambientales (y, por lo tanto, sociales) terminaron con la vida de mi madre: incluso aunque ella no fue maestra rural ni vivió nunca en áreas agrícolas seguramente se encontró expuesta a contaminantes ambientales de diverso tipo. Yo también había preferido “*no hacer cadenas*” y había dejado que una serie de narrativas hegemónicas despolitizadoras del cáncer e invisibilizadoras de sus causales me ofrecieran interpretar su padecimiento -y, por lo tanto, el mío- en clave individual: como el producto de los traumas psicológicos de mi madre, de sus malos hábitos de salud individual o de su carga genética. 

La investigación centrada en las maestras rurales y sus padecimientos en torno al cáncer me permitió entonces reflexionar sobre mi propia historia y sobre las limitaciones ante las que me he encontrado para pensarla en términos sociales. Me devolvió, en definitiva, interrogantes sobre mi propia realidad cultural y en parte de eso se trata el ejercicio de hacer etnografía: no solo de preguntar cómo y por qué los sujetos se formulan y responden preguntas sobre sus vidas -o no lo hacen- sino también de permitir que el análisis de esas otras realidades transforme nuestra propia manera de pensar, de mirar y de ser^47^. Una transformación que nos permita pensar, como en este caso, cómo y por qué nos formulamos y respondemos -o no formulamos ni respondemos- ciertas preguntas cruciales acerca de nuestra propia existencia. 

La historia de Lidia -y de las luchas que han dado las vecinas y los vecinos de su localidad y la de tantos otros pueblos “fumigados” de la pampa húmeda- evidencia que los padecimientos asociados al cáncer pueden dejar de estar circunscriptos al ámbito privado y de vivirse como tragedia individual o como prueba personal que hay que dar (para merecer la vida) cuando se los elabora en términos de denuncia, resistencia o lucha colectiva. Es también en esa clave colectiva -o al menos en el encuentro de una investigadora con sus entrevistadas o de la autora de este artículo con sus posibles lectoras y lectores- que la historia personal de una pérdida se relee en clave social y que un listado de nombres sueltos y desarticulados puede transformarse en la prueba y el retrato de una problemática sanitaria sobre la que formular, al menos, nuevas preguntas e investigaciones hasta llegar a las raíces de sus causas verdaderas. En esos grandes o pequeños procesos reside -con mayor o menor grado de sistematicidad y eficacia- la evidencia de la agencia humana, a pesar de los intentos hegemónicos de invisibilización de la enfermedad y de aquello que la provoca. 

## REFLEXIONES FINALES

A lo largo de estas páginas he buscado problematizar la relación entre la exposición ocupacional a agroquímicos y la existencia de cáncer entre personas que trabajan como docentes rurales. He indagado esta relación a propósito de un universo empírico particular: el de las maestras rurales de la zona sudeste de la provincia de Córdoba (Argentina) caracterizada por la producción de soja y maíz transgénicos tratados de manera intensiva con plaguicidas agrícolas. 

En la confluencia entre los materiales construidos a partir de un trabajo de campo antropológico realizado en la zona, los aportes formulados por otros estudios sobre el tema y las contribuciones de una perspectiva teórica particular, he propuesto, en primer lugar, que existen procesos sociales que tienden a invisibilizar la posible relación entre la exposición ocupacional a los agroquímicos y la existencia de cáncer. Así, la falta de información epidemiológica, el ocultamiento de datos anteriormente existentes o el subregistro de enfermedades ocupacionales docentes y de aquellas vinculadas con la exposición a agroquímicos abonan una narrativa hegemónica para la cual, la salud de la docencia rural expuesta a agroquímicos aparece, más bien, silenciada o invisibilizada. 

He evidenciado, además, cómo algunos de los elementos centrales de esa narrativa se encuentran entramados en los sentidos cotidianos de las docentes rurales acerca de la asociación agroquímicos-cáncer. Aun reconociendo su exposición ocupacional a los plaguicidas químicos y la existencia de numerosos casos de cáncer en sus comunidades, no consideran que ambos elementos puedan asociarse: diferentes voces autorizadas y argumentos dominantes que analicé críticamente las invitan a no indagar en esta posible vinculación. Sus sentidos, entonces, no pueden ser pensados en abstracto y por fuera de la existencia de esos procesos de invisibilización que van desde operaciones de silenciamiento hasta lenguajes “pedagógicos” más activos difundidos por los sectores agrícolas dominantes.

En un segundo momento propuse que, a pesar de estos procesos de invisibilización, la reconstrucción de las historias de vida de las docentes a partir de las entrevistas en profundidad evidenció que, si bien no son sus propios cuerpos los que padecen el cáncer, sus vidas sí se encuentran en gran medida atravesadas por padecimientos vinculados a esta enfermedad: son maestras que han enviudado prematuramente por cáncer; son maestras huérfanas por cáncer; son maestras que han perdido a personas del vecindario, amigas y también compañeras de trabajo por cáncer. Respecto de este último punto, fue posible reconstruir un listado de nombres propios de maestras rurales de la zona enfermas o fallecidas por cáncer y casi todas ellas afectadas particularmente por cáncer de mama (un tipo de cáncer que ha sido vinculado científicamente con la exposición a plaguicidas).

A propósito de este listado “artesanal” planteé que los nombres allí enlistados -y el tipo de cáncer mayoritario que habían padecido- me conectaron con mi historia personal como hija de una mujer fallecida prematuramente a causa de un cáncer de mama. La recuperación de mi propia experiencia en relación con ese evento me permitió reflexionar, en primer lugar, acerca del sufrimiento subjetivo que se condensa en torno al nombre propio de cada maestra enferma o fallecida por cáncer. Pero también me invitó a objetivar las dificultades y condicionamientos sociales que se interponen en el camino de la interpretación de esa vivencia traumática personal como un hecho con causas sociales y ambientales que deben ser indagadas. Concluí que, a partir de historias de demanda y lucha colectiva como la protagonizada por la docente Lidia junto con su comunidad, es que esos sucesos personales dejan de ser “maldiciones personales” para pasar a ser leídos en clave social. 

Mientras escribo estas líneas, de hecho, se prepara en el partido de Exaltación de la Cruz (provincia de Buenos Aires) un festival destinado a denunciar la criminalización de la protesta ambiental luego de que tres activistas fueran procesados judicialmente por desplegar una bandera con la leyenda *“Basta de cáncer. Paren de fumigarnos”* durante un acto oficial en el que se encontraba Alberto Fernández, presidente de la Nación. Que la existencia del cáncer y su asociación con las fumigaciones con agroquímicos pueda ser enunciada como consigna política que se reivindica y denuncia ante la máxima figura de autoridad a nivel nacional nos invita a pensar que la esperanza de la que habla la maestra rural entrerriana Estela Lemes -en el epígrafe que he elegido para iniciar estas páginas- no es, en lo absoluto, una utopía vana.

## References

[1] Schmidt M, Toledo López V (2018). Agronegocio, impactos ambientales y conflictos por el uso de agroquímicos en el norte argentino. Revista Kavilando.

[2] Arancibia F (2020). Resistencias a la bio-economía en Argentina: las luchas contra los agrotóxicos (2001-2013). Ciencia Digna.

[3] Toledo López V, Pereyra H, García Battán J, Ceirano V (2020). Riesgos e impactos socio-sanitarios del uso de agroquímicos: un estudio de caso en Selva, Santiago del Estero, 1990-2019. Revista Argentina de Salud Pública.

[4] Berger M, Carrizo C, Merlinsky G (2020). Cartografías del conflicto ambiental en Argentina III.

[5] Red Federal de Docentes por la Vida (2019). Por una escuela libre de tóxicos.

[6] Caisso L (2021). La lucha socioambiental en las aulas, el legado de Ana Zabaloy.

[7] Confederación de Trabajadores de la Educación de la República Argentina (2019). Protocolo de actuación ante un caso de aplicación con agroquímicos en las adyacencias de establecimientos educativos.

[8] Diez C (2016). El ojo en el veneno: ambientalización de los conflictos en la producción agropecuaria en Misiones a partir del caso tabacalero. Kula.

[9] Lucero P (2019). “Fumigado o no fumigado, todos los días me voy al campo”: Etnografía sobre los sentidos nativos del riesgo de enfermar por agrotóxicos en Morse, provincia de Buenos Aires.

[10] Kunin J, Lucero P (2020). Percepción social del riesgo y dinámicas de género en la producción agrícola basada en plaguicidas en la pampa húmeda argentina. Sexualidad, Salud y Sociedad.

[11] Caisso L (2017). ¿Una temática “en boga” o un problema silencioso?: Primeras reflexiones sobre el abordaje etnográfico de una problemática socioambiental en una localidad rural (Córdoba, Argentina). Runa.

[12] Caisso L (2022). Escuelas rurales, docentes y fumigaciones con agroquímicos: Del registro del silenciamiento social al registro de las resistencias cotidianas. Cuadernos de Antropología Social.

[13] Evia V (2018). Saberes y experiencias sobre la exposición a plaguicidas entre mujeres que residen en contextos agrícolas en Soriano, Uruguay. Revista Trama.

[14] Evia V (2021). Venenos, curas y matayutos: Trabajadores agrícolas y saberes sobre plaguicidas en Uruguay. Revista de Ciencias Sociales.

[15] Kretschmer R, Areco A, Palau M (2020). Escuelas rurales fumigadas en Paraguay: Estudio de tres casos en tres distritos.

[16] González DV (2022). Ambientalista soy yo: El bloque dominante en el conflicto por el uso de agrotóxicos en el partido de Pergamino (Buenos Aires, Argentina).

[17] Lapegna P (2019). La Argentina transgénica: De la resistencia a la adaptación, una etnografía de las poblaciones campesinas.

[18] Arancibia F, Motta R (2018). Undone science and counter-expertise: Fighting for justice in an Argentine community contaminated by pesticides. Science as Culture.

[19] Löwy C (2019). La construcción del discurso agroquímico plaguicida: De la OMS a los Territorios.

[20] Folguera G (2020). La ciencia sin freno: de cómo el poder subordina el conocimiento y transforma nuestras vidas.

[21] Gárgano C (2022). Mujeres con “La soja al cuello”: Experiencias y evidencias en torno a la contaminación de cuerpos y territorios en Buenos Aires. HALAC.

[22] Galt RE (2007). Regulatory risk and farmers’ caution with pesticides in Costa Rica. Transactions of the Institute of British Geographers.

[23] Kannuri N, Jadhav S (2018). Generating toxic landscapes: impact on well-being of cotton farmers in Telangana, India. Anthropology & Medicine.

[24] Bureau-Point E (2019). Pesticides uses and risk perceptions.

[25] Shattuk A (2021). Risky subjects: Embodiment and partial knowledges in the safe use of pesticide. Geoforum.

[26] Grandia L (2022). Poisonous exports: Pesticides, peasants, and conservation paradigms in Guatemala. Latin American Perspectives.

[27] Álvarez MFS (2009). Pocos ganan, muchos pierden: soja, agroquímicos y salud: Departamento Río Segundo, provincia de Córdoba, Argentina.

[28] Pujol C (2012). La relación plaguicidas-salud-ambiente en un caso testigo: Isla Verde, Provincia de Córdoba.

[29] Avila Vazquez M, Ruderman L, Maturano E, Maclean B, Difilippo F, Marino D, Andrinolo D Evaluación de la salud colectiva socio-ambiental de Monte Maíz.

[30] Delgado F (2020). Prácticas agrícolas intensivas, salud y población: Autopercepción acerca del riesgo.

[31] Verseñazzi D, Vallini A, Fernández F, Ferrazini L, Lasagna M, Sosa A, Hough G (2023). Cancer incidence and death rates in Argentine rural towns surrounded by pesticide-treated agricultural land. Clinical Epidemiology and Global Health.

[32] Peralta P, Mañas F, Gentile N, Bosch B, Méndez A, Aiassa D (2011). Evaluación del daño genético en pobladores de Marcos Juárez expuestos a plaguicidas: estudio de un caso en Córdoba. Revista Diálogos.

[33] Butinof M, Fernández R, Muñoz S, Lerda D, Blanco M, Lantieri MJ (2017). Valoración de la exposición a plaguicidas en cultivos extensivos de Argentina y su potencial impacto sobre la salud. Revista Argentina de Salud Pública.

[34] Garassini ME (2015). Narrativas de familiares de pacientes con cáncer. CES Psicología.

[35] Suárez-Rienda V, López Sánchez O (2019). La dimensión emocional en torno al cáncer: Estrategias de análisis desde la antropología de la salud. Cuicuilco, Revista de Ciencias Antropológicas.

[36] Porroche Escudero A (2019). Elementos para la despolitización del cáncer de mama. Atlánticas, Revista Internacional de Estudios Feministas.

[37] Passerino L (2020). Experiencia y finitud: un abordaje fenomenológico en mujeres que transitan cáncer de mama en el Área Metropolitana de Buenos Aires (Argentina). Revista M.

[38] Passerino L (2019). Cuidado de sí y experiencia de enfermedad: Aportes desde una perspectiva de género al tránsito de mujeres con cáncer de mama en el área metropolitana de Buenos Aires. Cuadernos de la Facultad de Humanidades y Ciencias Sociales.

[39] Crehan K (2004). Gramsci, cultura y antropología.

[40] Crehan K (2018). El sentido común en Gramsci: La desigualdad y sus narrativas.

[41] Roseberry W, Lagos M, Calla P (2004). Antropología del Estado: Dominación y prácticas contestatarias en América Latina.

[42] Menéndez E (2008). Epidemiología sociocultural: propuestas y posibilidades. Región y Sociedad.

[43] Menéndez E (2022). Relaciones sociales y procesos de salud/enfermedad: las razones y los hechos. Cuadernos de Antropología Social.

[44] Baer HA (1996). Toward a political ecology of health in medical anthropology. Medical Anthropology Quarterly.

[45] Menéndez E (2005). El modelo médico y la salud de los trabajadores. Salud Colectiva.

[46] Haro JA (2010). Epidemiología sociocultural: Un diálogo en torno a su sentido, métodos y alcances.

[47] Rockwell E (2009). La experiencia etnográfica: Historia y cultura en los procesos educativos.

[48] Ramírez Hitta S (2009). La contribución del método etnográfico en el registro del dato epidemiológico. Salud Colectiva.

[49] Yannoulas S (1996). Educar: ¿una profesión de mujeres?: la feminización del normalismo y la docencia: 1870-1930.

[50] Morgade G, Morgade G (1997). Mujeres en la educación: Género y docencia en la Argentina 1870-1930.

[51] Soto G, Cabrol D, Seifert S, Aguila Wharton A, Taranda N, Tiscornia L (2020). Estructuras agrarias provinciales con datos censales y fuentes alternativas. Realidad Económica.

[52] Guber R (2001). La etnografía, método, campo y reflexividad.

[53] Achilli E (2005). Investigar en antropología social: Los desafíos de transmitir un oficio.

[54] Pórfido O (2013). Los plaguicidas en la República Argentina.

[55] Gobierno de la Provincia de Córdoba, Ministerio de Salud (2017). Cáncer: incidencia y mortalidad en córdoba 2004-2013, Provincia de Córdoba y Departamento Capital.

[56] La Voz (2014). El mapa del cáncer en Córdoba. La Voz.

[57] Barri F (2014). Soja, ambiente y salud: debates pendientes en relación al actual modelo de desarrollo para el campo argentino. Voces en el Fénix.

[58] Sández F (2019). La argentina fumigada: Agroquímicos, enfermedad y alimentos en un país envenenado.

[59] Superintendencia de Riesgos del Trabajo (2012). Accidentabilidad laboral en sectores específicos de la economía.

[60] Superintendencia de Riesgos del Trabajo (2006). Panorámica de los riesgos laborales en el sector agrario: informe especial.

[61] Dubois D, Cuesta F (2021). Estela Lemes: “Decirme a mí que tengo razón, es decirle al pueblo argentino que los agroquímicos enferman”.

[62] Lucero P (2022). El silencio no es salud: Prácticas y discursos sobre los profesionales de la salud en el partido de Junín (provincia de Buenos Aires) sobre el uso de agrotóxicos en la agricultura extensiva entre 2015 y 2018. Cuadernos de Antropología Social.

[63] Caisso L, Carreño G (2022). La Ley de Buenas Prácticas Agropecuarias de la provincia de Córdoba: Un análisis a propósito de la problemática de las fumigaciones con agroquímicos. Pampa.

[64] Santamaría-Ulloa C (2009). El impacto de la exposición a plaguicidas sobre la Incidencia de Cáncer de mama. Evidencia de Costa Rica. Población y Salud en Mesoamérica.

[65] Ventura C, Venturino A, Miret N, Randi A, Rivera E, Núñez M, Cocca C (2015). Chlorpyrifos inhibits cell proliferation through ERK1/2 phosphorylation in breast cancer cell lines. Chemosphere.

[66] Kass L, Gomez A, Altamirano G (2020). Relationship between agrochemical compounds and mammary gland development and breast cancer. Molecular and Cellular Endocrinology.

[67] Lasagna M, Ventura C., Hielpos M S, Mardirosian M N, Martín G, Miret N, Randi A, Núñez M, Cocca C (2022). Endocrine disruptor chlorpyrifos promotes migration, invasion, and stemness phenotype in 3D cultures of breast cancer cells and induces a wide range of pathways involved in cancer progression. Environmental Research.

